# Relationship between automated choroidal thickness measurements and retinal sensitivity using microperimetry in patients with myopia and different stages of myopic maculopathy

**DOI:** 10.1186/s40942-024-00541-9

**Published:** 2024-03-08

**Authors:** Fillipe de Biaggi Borges da Silva, Luis Claudio Pimentel Silva, Leonardo Provetti Cunha, Leandro Cabral Zacharias, Eduardo V. Navajas, Mario L. R. Monteiro, Rony C. Preti

**Affiliations:** 1 Division of Ophthalmology, University of São Paulo Medical School, São Paulo, São Paulo Brazil; 2 Division of Ophthalmology, Federal University of Juiz de Fora, Minas Gerais, Brazil; 3 Division of Ophthalmology, University of British Columbia, Vancouver, Canada

**Keywords:** Optical coherence tomography, Myopia, High myopia, Retinal sensitivity, Microperimetry, Pathologic myopia

## Abstract

**Purpose:**

To assess the relationship between macular choroidal thickness (CT) measurements and retinal sensitivity (RS) in eyes with myopia and different stages of myopic maculopathy.

**Methods:**

A masked, cross-sectional, and consecutive study involving patients with emmetropia/myopia (control group) and high myopia (HM) eyes. Automated choroidal thickness (CT) and manual outer retinal layer (ORL) thickness were acquired using swept-source optical coherence tomography, while retinal sensitivity (RS) assessed by microperimetry (MP3) in all regions of the macular Early Treatment Diabetic Retinopathy Study (ETDRS) grid. Comparisons were made between groups, and correlations were performed among these measurements, demographic and ocular parameters and myopic maculopathy classification.

**Results:**

A total of 37 (74 eyes) patients were included in the study. The mean age was 39 ± 13 years, and 28 patients (76%) were female. HM eyes exhibited inferior best-corrected visual acuity and a more advanced myopic maculopathy classification compared to the control group. The mean macular CT were 255 and 179 μm in the control and HM eyes (P < 0.001), respectively. In the HM eyes, superior ETDRS region presented the greatest values. Mean RS in control and HM groups was 28 and 24 dB (P = 0.001), respectively. Inner temporal followed by superior, were the regions of higher RS. Mean ORL thickness was 83 and 79 μm (P < 0.001), in the control and HM groups, respectively. The inner temporal ETDRS region presented the thickest measure. CT correlated significantly with RS (r = 0.41, P < 0.001) and ORL thickness, (r = 0.58, P < 0.001), which also correlated with RS (r = 0.40, P < 0.001). Spherical equivalent, axial length and myopic maculopathy stage were the parameters that most correlated with CT, RS and ORL thickness. For every 100 μm increase in thickening of CT there was an average increase of 3.4 μm in ORL thickness and 2.7 dB in RS. Myopic maculopathy classification demonstrated influence only with CT.

**Conclusion:**

Myopia degree is related to ORL and choroidal thinning and deterioration of retinal sensitivity in some ETDRS regions of the macula. Choroidal thinning is associated to with a decline of retinal sensitivity, thinning of ORL, and worsening of myopic maculopathy classification, so new treatments are necessary to prevent myopia progression.

## Introduction

The prevalence of myopia is escalating rapidly, rendering it a significant public health concern owing to its potential impact on vision loss [1]. This is attributable to the histological alterations observed in both the retinal tissue, crucial for vision, and the choroid, which appears to exert a pivotal role in pathological conditions affecting the macula [2, 3]. The augmentation of the postero-anterior diameter in highly myopic (HM) (spherical equivalent objective refractive error > − 6.0 diopters) eyes in addition to inducing retinal and choroidal thinning [4, 5], enhances the susceptibility to various conditions frequently associated with vision impairment. These conditions encompass retinal detachment, macular holes, foveoschisis, chorioretinal atrophy, choroidal neovascularization [6], and, in some instances, optical neuropathy [7].

Optical coherence tomography (OCT) serves as a valuable diagnostic tool for detecting and precisely measuring retinal and choroidal changes at a micrometer scale. In the clinical assessment of various macular diseases, the macular grid recommended by the *Early Treatment Diabetic Retinopathy Study* (ETDRS) is commonly used to reference the location of observed damage in OCT scans [8, 9]. However, despite its anatomical insights, OCT lacks the capability to conduct functional analyzis of the retina. Consequently, a direct comparison between anatomical changes and visual function remains unattainable.

Over the years, the measurement of retinal sensitivity (RS) has been conducted through computerized perimetry. However, with technological advancements, studies employing the microperimeter (MP) have gained prominence for this purpose [10]. The MP facilitates analyzes comparable to traditional Humphrey automated perimeter visual field test, with the added benefit of a system capable of real-time readjustment to eye movements. This feature enables the examination of specific points of RS within a direct correlation within the macular defined region, concurrently allowing the capture of fundus imaging [11].

Qin et al. demonstrated a reduction in macular light sensitivity correlating with the severity of myopia [12]. However, their study did not establish a correlation between retinal and choroidal structural changes to elucidate this finding, and it utilized the previous commercially available technology, the MP1 (ver. SW1.4.1 SP1; Nidek Technologies). In 2015, Zaben et al. identified a direct, inverse correlation between RS and CT exclusively in a group of highly myopic (HM) patients [13]. While their results were intriguing, showcasing the lowest RS at the center of the macula, limitations include the absence of a control group and manual choroidal measurements. Furthermore, none of the aforementioned studies explored the relationship between RS and the classification of myopic maculopathy, nor did they establish a direct topographic correlation using the ETDRS chart with RS, CT, and outer retinal thickening concurrently.

So, the objective of this study was to measure choroidal thickness (CT), outer retinal layer (ORL) thickness, and macular retinal sensitivity (RS) assessed by microperimetry (MP), and to examine their correlations in eyes with myopia. Additionally, the study aimed to assess the association of these measurements with different stages of myopic maculopathy.

## Methods

This masked, cross-sectional, observational, and descriptive study adhered to the principles outlined in the Declaration of Helsinki and received approval from the Institutional Review Board Ethics Committee at the University of São Paulo Medical School, São Paulo, Brazil. Informed consent was obtained from all participants prior to their enrollment.

The study included 37 patients categorized into two groups, each with a best-corrected visual acuity (BCVA) of  ≥ 20/200. The criteria used to assign subjects to control or HM group, was the spherical equivalent objective refractive error. The control group, comprising 44 eyes, included emmetropes (spherical equivalent objective refractive error ranging from + 0.50 to − 0.50) and myopic individuals (spherical equivalent objective refractive error ranging from − 0.50 to − 6.00). The second group, consisting of 30 eyes, comprises individuals with HM (spherical equivalent objective refractive error greater than − 6.00). Exclusion criteria were a history of prior intraocular surgery, pregnancy, glaucoma, retinopathies, diseases falling within the pachychoroid spectrum, uveitis, presence of systemic conditions such as Cushing’s syndrome, kidney diseases, rheumatologic disorders, use of corticosteroids, and significant fixation deficits that could impede proper OCT evaluations or any other ocular disorders that could potentially compromise OCT evaluations. When eligible, both eyes of the same patient were included in the study.

### Ocular clinical examination

All participants underwent a comprehensive ocular examination, including best-corrected visual acuity (BCVA) assessment using the Early Treatment Diabetic Retinopathy Study (ETDRS) table at 4 m, with logMAR conversion. Indirect binocular ophthalmoscopy and posterior segment biomicroscopy were conducted under mydriasis, utilizing a 20-diopter lens and a 90-diopter lens (Volk Lens^®^), respectively. Fundoscopic findings led to the categorization of patients into categories 0, 1, 2, 3, and 4, in accordance with the international photographic classification and categorization system for myopic maculopathy [14]. Following the clinical evaluation, patients underwent additional assessments, including axial length measurement using the IOL Master (Carl Zeiss, Jena, Germany), microperimetry with the MP3 device (NIDEK Co. Ltd., Aichi, Japan), and optical coherence tomography (OCT) with the DRI OCT Triton plus^®^ (TOPCON^®^).

Participants were instructed to refrain from consuming products containing alcohol, nicotine, and caffeine 5 h before the exams. Additionally, they were advised to limit their liquid intake for a period of 6 h preceding the test [15, 16]. All the exams were done in the morning.

### OCT analysis of the choroid

**Fig. 1:**
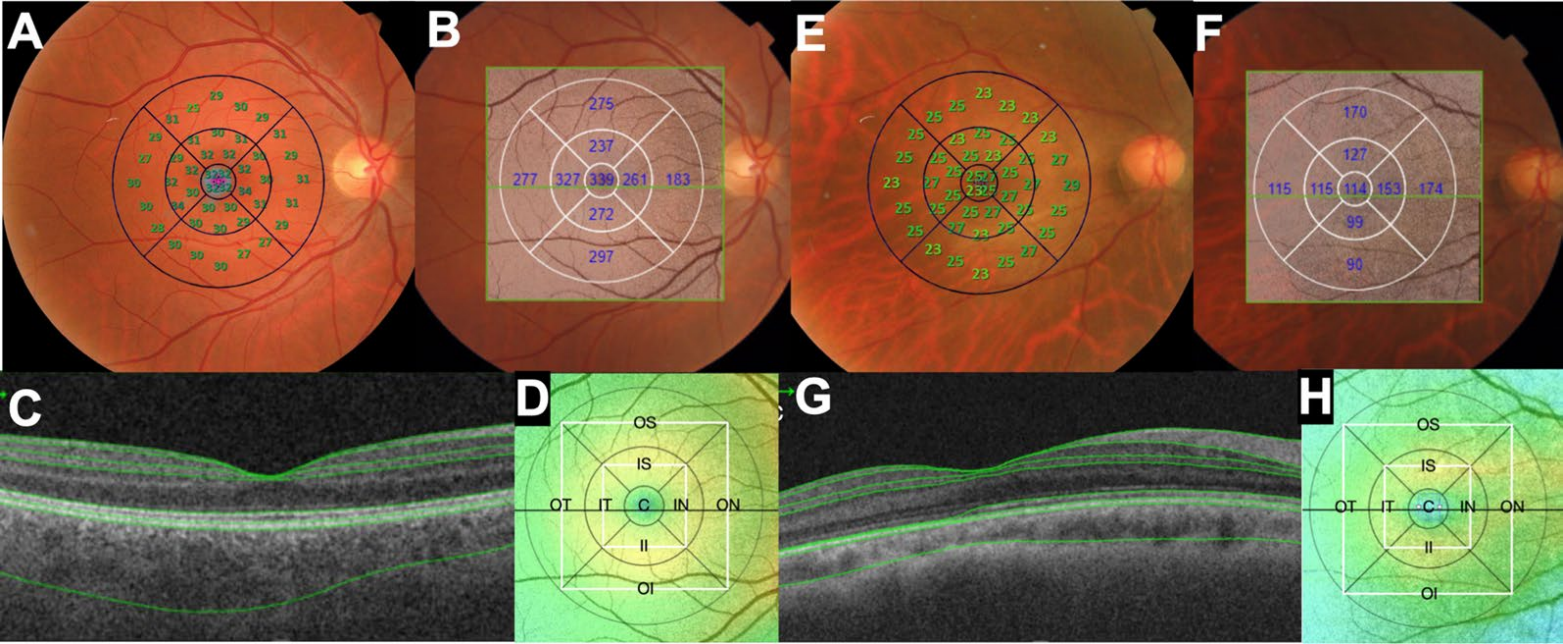
**A**–**D** Representative images of the control group of a patient with 28 year-old with BCVA 0 logMAR, axial length 24.84 mm, spherical equivalent of − 0.50 diopters and central ORL thickness of 87.11 µm. **A** retinal sensitivity (RS) plotted over the macula; **B** choroidal thickness at each ETDRS regions; **C** retinal and choroid automated layers segmentation and **D** choroidal thickness topographic map. **E**–**H** Representative images of the high myopic group of a patient with 43 year-old with BCVA 1 logMAR, axial length 30.43 mm, spherical equivalent of − 19.00 diopters and central ORL thickness of 76.55 µm. **E** RS plotted oover the macula; **F** choroidal thickness at each ETDRS regions; **C** retinal and choroid automated layers segmentation and **D** choroidal thickness topographic map.** C**, central; SI, superior inner; TI, temporal inner; II, inferior inner; NI, nasal inner; OS, outer superior; outer temporal; OI, outer inferior; ON, outer nasal

A blinded investigator employed the 3D Macula protocol available on the equipment. This protocol conducts a horizontal scan in the macular area covering 7 × 7 mm with a scan density of 512 × 256. The images were acquired using the device's internal fixation under mydriasis, facilitating the generation of a 3D image and providing a choroidal ETDRS grid–grid thickness map. Before automated software analysis, the automated segmentation was meticulously reviewed. The selected reference line for measurement was the posterior border of the retinal pigment epithelium (RPE) and the anterior surface of the sclerochoroidal junction, used to derive choroidal thickness (CT). In the event of a segmentation error, corrective measures were applied, and the images were subsequently processed for measurement acquisition, as depicted in Fig. 1.

### Outer retinal layer thickness

**Fig. 2:**
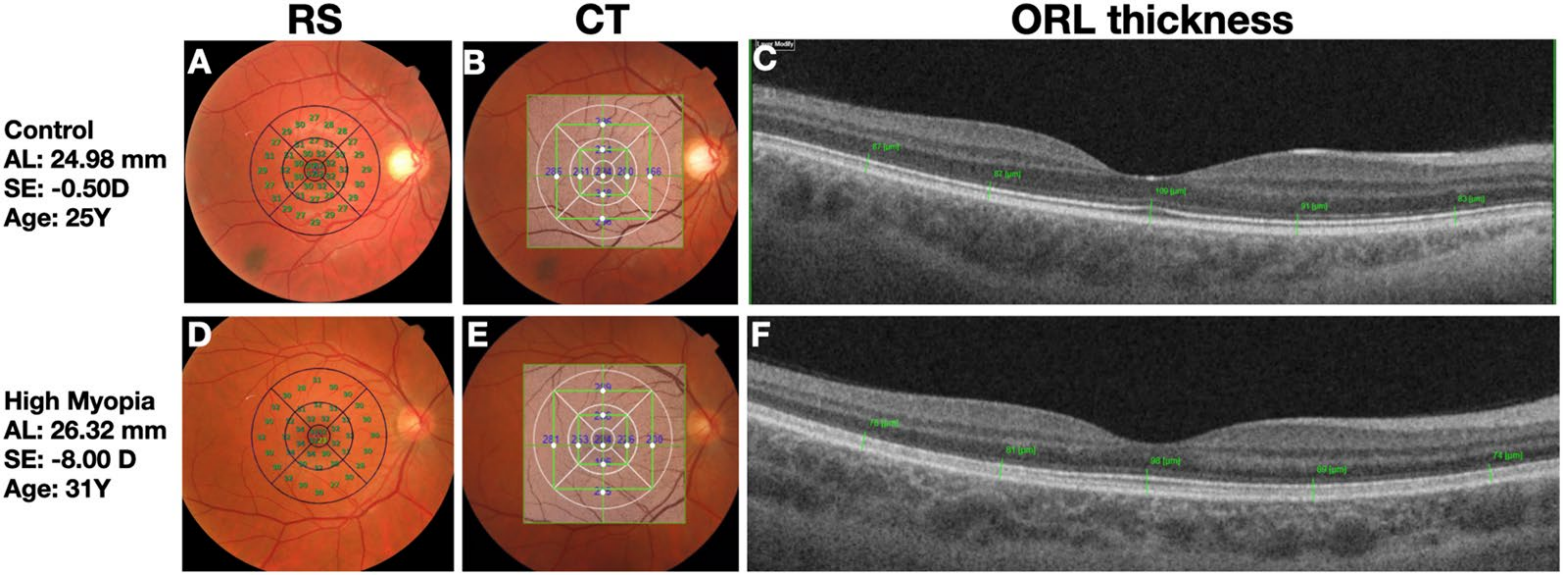
Representative microperimetry, retinal sensitivity (RS), choroidal thickness (CT), and outer retinal layer (ORL) thickness images of control and high myopia (HM) groups. **A** and **D**, fundus photographic image with RS assessed via microperimetry (MP3). **B** and **E**, fundus photographic image depicting CT in each Early Treatment Diabetic Retinopathy Study (ETDRS) region and the points where the ORL was measured. **C** and **F**, B-scan image demonstrating the measurement of ORL thickness, obtained at 9 spots according to ETDRS standards, from the external limiting membrane to the inner edge of RPE/Bruch membrane

The thickness of the outer retinal layers (ORL), delineated by the inner limit of the external limiting membrane and the outer edge of the retinal pigment epithelium (RPE), was manually measured using the device’s caliper. The ETDRS grid consists of concentric and radial lines, forming inner, middle, and outer rings. At the intersections of these lines in the upper and lower ETDRS regions, a horizontal line was drawn, and the thickness was measured at the intersection of a vertical line passing through the fovea. Conversely, in the temporal and nasal macular regions, a vertical line was drawn from the intersection of ETDRS grid lines, and the ORL thickness was obtained at the intersection point where a horizontal line passing through the fovea reach these vertical lines, as illustrated in Fig. 2.

### Microperimetry test

RS assessment was conducted with MP-3 microperimeter (Nidek Co., Ltd., Aichi, Japan) device in all patients. The MP stimulus employed in this study utilized a Goldmann size III stimulus, projected for a duration of 200 ms. This stimulus presented against a white-on-white background with a luminance of 1.27 CD/m^2^, equivalent to 4 apostilb (ASB). The maximum luminance achievable by MP stimulus was set at 10,000 ASB, and the stimulus attenuation light could be adjusted within a range spanning from 0 dB, signifying the maximum stimulus luminance to 34 dB, signifying the lowest stimulus luminance achievable. Notably, an area unable to perceive the maximum visual stimulus threshold, was categorized as an ‘absolute scotoma’ and assigned a value of 0 dB. To assess RS, a 4–2 threshold strategy, known as the ‘Full-Threshold Staircase’ was implemented. Additionally, the MP test incorporates an eye-tracking system to compensate for ocular movements and monitor fixation stability. All exams were performed after pupil dilation.

The patients’ spherical equivalents were not used to adjust the image sharpness because the RS assessment technique permits automatic recognition, automatically adjusting the range of focus between − 25 diopter (D) and 15 D for the right eye/left eye. In our studies we had one patient with myopia − 26 D, that the exam was based on their contact lenses.

The testing protocol commenced with a preliminary phase, involving two consecutive MP tests, aimed in familiarizing the patient and enhancing the reliability of subsequent assessment. Following this, a 15 min resting period was observed, followed by a 5 min mesopic adaptation period before the main examination.

During the examination, the device’s software automatically computed the mean average of the threshold measurements, expressed in dB, from all 44 test points. This average corresponded to the global mean RS in dB. Moreover, mean RS responses were separately determined for each of the nine regions delineated in the ETDRS map.

To facilitate patient fixation, a 4°degree red cross served as the target. This comprehensive MP testing approach aimed to provide a robust assessment of RS while accounting for factors such as eye movement and fixation stability.

The MP test involved the evaluation of MP parameters across a grid comprising 44 test points, encompassing a total of 20°degrees at the macular region. This grid spanned (10°degrees from the center of the fovea in both horizontal directions) and corresponded directly to a 6 mm in diameter region at the macular area. Importantly, this grid exhibited a direct topographic alignment with the nine regions of the ETDRS map (Fig. 1).

This direct topographic correspondence allowed for a precise correlation between RS-tested points on the MP grid and the measurements CT (in µm) within the nine regions of the ETDRS map. Specifically, each region on the inner, middle and outer rings of the ETDRS map as covered by the OCT scans encompassed five RS tested points. In contrast, the central circle with 1 mm diameter (also represented on Fig. 1), featured four RS-tested points for assessment. The mean RS in each at 9-ETDRS grid region was then calculated.

### Statistical analysis

The ages and genders of patients were delineated using summary measures, including mean, standard deviation (SD), median, and quartiles, along with absolute and relative frequencies. Parameters assessed in the eyes and macular ETDRS regions were outlined based on eye groups, utilizing means and SD. Group and BCVA comparisons were conducted through Generalized Estimating Equations (GEE) with normal distribution. For the classification of myopic maculopathy, comparisons were made with GEE employing Poisson distribution. In all analyses, an exchangeable correlation matrix was assumed between the eyes of the same patient.

Spearman correlations between personal and ophthalmic characteristics with parameters of retinal ETDRS regions were calculated. Myopic maculopathy models were developed for each macular parameter using GEE with Poisson distribution to examine their relationship within the myopic maculopathy. Additionally, models were created for each macular parameter (RS, CT and ORL thickness) incorporating personal and ophthalmic characteristics, utilizing GEE with normal distribution.

All analyzes were performed using IBM-SPSS for Windows version 20.0, and results were tabulated using Microsoft Excel 2003. Statistical tests were conducted with a significance level of 0.05%.

## Results

The mean age was 39 ± 13 years (female 39 ± 13 and male 42 ± 17) and 28 patients (76%) were female. HM eyes exhibited inferior BCVA, greater spherical equivalent (SE), increased axial length (AL), and a more advanced myopic maculopathy classification compared to the control group, as presented in Table 1.

**Table 1:** The comparison of ocular demographic data between the two groups

Variable	Group		p	p*	p^sex^	p^age^
	Control	HM				
BCVA						
Mean ± SD	0.38 ± 0.48	0.18 ± 0.31	**0.017**	** < 0.001**	0.24	** < 0.001**
Median (p25; p75)	0 (0; 1)	0 (0; 0.18)				
Spherical equivalent						
Mean ± SD	− 1.1 ± 1.9	− 11.4 ± 5.6	** < 0.001**	** < 0.001**	0.509	0.213
Median (p25; p75)	− 0.5 (− 1.8; 0)	− 8.5 (− 13.6; − 7.7)				
Axial length (mm)						
Mean ± SD	24.3 ± 1	27.7 ± 1.9	** < 0.001**	** < 0.001**	0.703	0.954
Median (p25; p75)	24.3 (23.4; 24.6)	27.4 (26.3; 28.5)				
Myopic maculopathy. n (%)						
No macular lesions	1 (2.3)	0 (0)	** < 0.001**	**0.001**	0.982	0.766
Tesselated fundus	42 (95.5)	0 (0)				
Difuse chorioretinal atrophy	1 (2.3)	30 (100)				

Bold values represent the best statistically significant results (p < 0.05)

*EEG* with normal distribuition

*p adjusted for sex and age

**Fig. 3:**
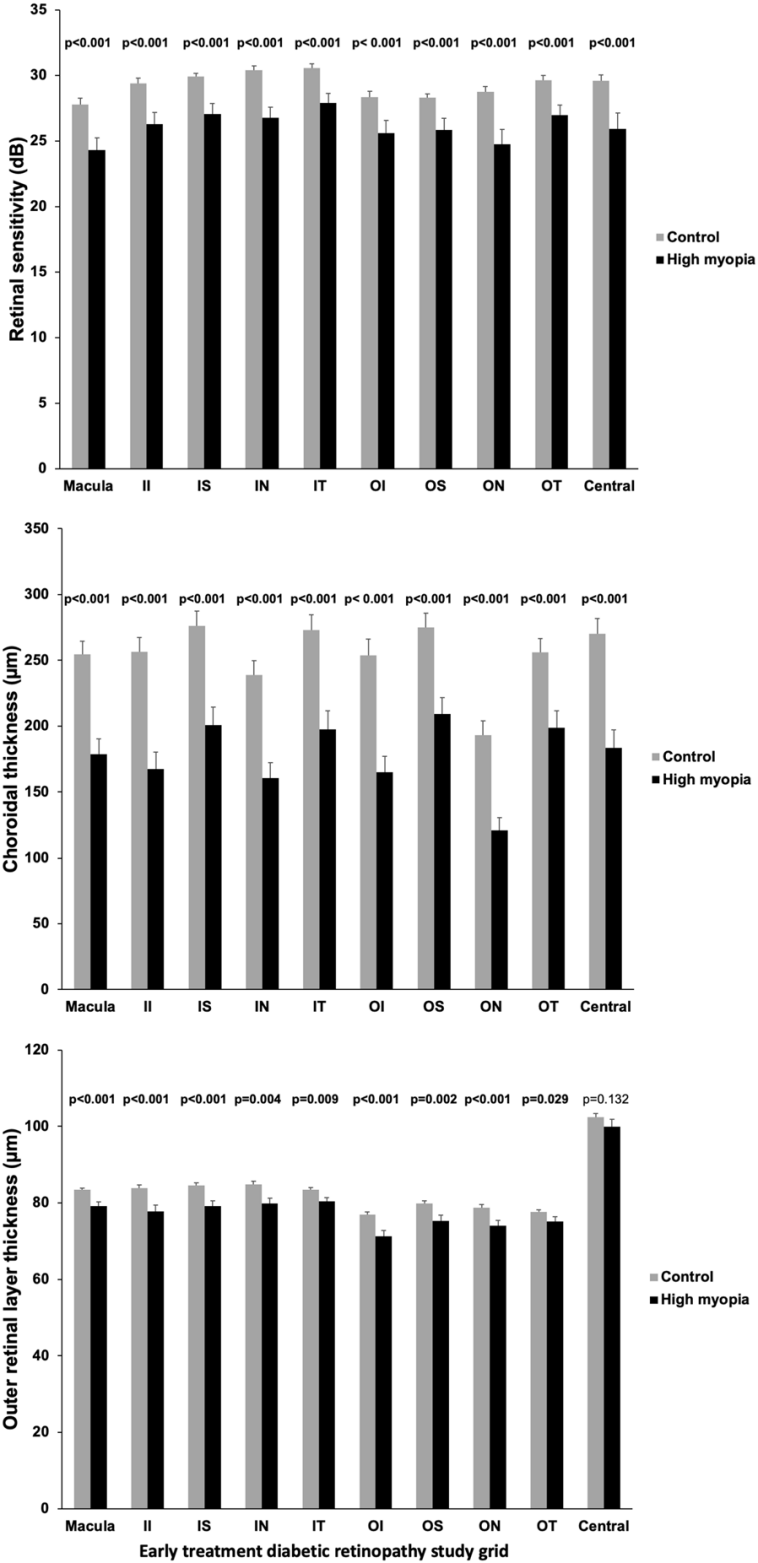
Graphic showing between groups comparison of retinal sensitivity, choroidal thickness and outer retinal layer thickness per ETDRS macular region between groups

The mean CT ETDRS macula ring, inferior inner, superior inner; NI, nasal inner, temporal inner, inferior outer, superior outer, nasal outer, temporal outer and central ring were: 254.7 ± 66, 256.4 ± 73.3, 276.2 ± 74.9, 238.7 ± 72.1, 273 ± 76.4, 253.7 ± 81.6, 275.1 ± 71.1, 193.3 ± 70.6, 256 ± 69.2, 270.4 ± 75.1 and 178.8 ± 63.4, 167.3 ± 71.5, 200.9 ± 74.8, 160.6 ± 63.9, 197.4 ± 78.4, 165.1 ± 66, 209.2 ± 67.8, 121.1 ± 52.4, 198.8 ± 70.1, 183.4 ± 75, from the control and HM groups respectively, a statistically significant difference, p < 0.001. Within the HM group, the superior inner ETDRS region exhibited the highest CT, followed by the superior outer region, when compared to other regions, Fig. 3 and Table 2. Figure 1 illustrates a unique case of HM eyes with nasal ETDRS region with a thicker choroid compared to other regions, despite being the region with the lowest value in the overall sample.

**Table 2:** Comparison of choroidal thickness, outer retinal layers thickness and retinal sensitivity between the control and high myopic groups

Variable	Group		p	p*	p^Sex^	p^age^
	Control	HM				
Choroidal thickness (µm)						
Macula (mean ± SD)	254.7 ± 66	178.8 ± 63.4	** < 0.001**	** < 0.001**	0.26	**0.023**
II	256.4 ± 73.3	167.3 ± 71.5	** < 0.001**	** < 0.001**	0.113	**0.044**
SI	276.2 ± 74.9	200.9 ± 74.8	** < 0.001**	** < 0.001**	0.252	0.067
NI	238.7 ± 72.1	160.6 ± 63.9	** < 0.001**	** < 0.001**	0.766	0.087
TI	273 ± 76.4	197.4 ± 78.4	** < 0.001**	** < 0.001**	0.225	**0.047**
IO	253.7 ± 81.6	165.1 ± 66	** < 0.001**	** < 0.001**	0.103	**0.019**
SO	275.1 ± 71.1	209.2 ± 67.8	** < 0.001**	** < 0.001**	0.42	**0.026**
NO	193.3 ± 70.6	121.1 ± 52.4	** < 0.001**	** < 0.001**	0.953	0.095
TO	256 ± 69.2	198.8 ± 70.1	** < 0.001**	** < 0.001**	**0.041**	**0.005**
Central	270.4 ± 75.1	183.4 ± 75	** < 0.001**	** < 0.001**	0.507	0.052
Retinal sensitivity (dB)						
Macula (mean ± SD)	27.8 ± 3.2	24.3 ± 5	**0.001**	** < 0.001**	0.567	** < 0.001**
II	29.4 ± 2.6	26.3 ± 4.8	** < 0.001**	** < 0.001**	0.25	** < 0.001**
SI	29.9 ± 1.8	27.1 ± 4.3	** < 0.001**	** < 0.001**	0.282	** < 0.001**
NI	30.4 ± 2.1	26.8 ± 4.4	** < 0.001**	** < 0.001**	0.341	** < 0.001**
TI	30.6 ± 2.2	27.9 ± 4.2	** < 0.001**	** < 0.001**	0.217	** < 0.001**
IO	28.3 ± 2.9	25.6 ± 5.3	**0.001**	** < 0.001**	0.193	** < 0.001**
SO	28.3 ± 1.7	25.9 ± 4.8	** < 0.001**	** < 0.001**	0.178	** < 0.001**
NO	28.8 ± 2.4	24.7 ± 6.3	** < 0.001**	** < 0.001**	0.543	** < 0.001**
TO	29.6 ± 2.3	27 ± 4.4	** < 0.001**	** < 0.001**	0.182	** < 0.001**
Central	29.6 ± 2.9	25.9 ± 6.5	** < 0.001**	** < 0.001**	0.655	** < 0.001**
ORL thickness (µm)						
Macula (mean ± SD)	83.4 ± 3.4	79.1 ± 6.4	**0.006**	** < 0.001**	0.112	**0.008**
II	83.9 ± 4.9	77.7 ± 9.4	**0.001**	** < 0.001**	0.43	0.085
SI	84.6 ± 4.7	79.2 ± 7	**0.001**	** < 0.001**	**0.021**	0.099
NI	84.8 ± 5.5	79.9 ± 7.5	**0.019**	**0.004**	0.336	**0.047**
TI	83.4 ± 3.9	80.4 ± 5.7	**0.016**	**0.009**	0.982	0.204
IO	77 ± 4.4	71.2 ± 8.6	**0.001**	** < 0.001**	0.794	0.076
SO	79.8 ± 4.9	75.3 ± 8.1	**0.017**	**0.002**	0.128	**0.009**
NO	78.8 ± 5.3	74.1 ± 7.3	**0.005**	** < 0.001**	0.335	**0.006**
TO	77.6 ± 4.1	75.1 ± 6.9	0.121	**0.029**	**0.044**	**0.038**
Central	102.5 ± 5.9	99.9 ± 10.6	0.32	0.132	**0.05**	**0.034**

EEG presenting normal distribuition

Bold values represent the best statistically significant results (p < 0.05)

*ORL* outer retinal layer, *HM* high myopic, *SI* superior inner, *TI* temporal inner, *II* inferior inner, *NI* nasal inner, *OS* outer superior, outer temporal, *OI* outer inferior, *ON* outer nasal

*p adjusted for sex and age

HM eyes demonstrated a lower mean RS measurement with MP3 at 25.9 ± 6.5 dB compared to 29.6 ± 2.9 dB in the control group. The inner temporal ETDRS region displayed the highest RS value, followed by inner superior ETDRS region, as detailed in Table 2 and Fig. 3. The mean ETDRS macula ring, inferior inner, superior inner, nasal inner, temporal inner, inferior outer, superior outer, nasal outer, temporal outer and central ring were: 27.8 ± 3.2, 29.4 ± 2.6, 29.9 ± 1.8, 30.4 ± 2.1, 30.6 ± 2.2, 28.3 ± 2.9, 28.3 ± 1.7, 28.8 ± 2.4, 29.6 ± 2.3, 29.6 ± 2.9 and 24.3 ± 5, 26.3 ± 4.8, 27.1 ± 4.3, 26.8 ± 4.4, 27.9 ± 4.2, 25.6 ± 5.3, 25.9 ± 4.8, 24.7 ± 6.3, 27 ± 4.4 and 25.9 ± 6.5, from the control and HM groups respectively.

The central ring ORL thickness was 102.5 ± 5.9 μm in control and 99.9 ± 10.6 μm in HM eyes, showing no statistically significant difference, p = 0.13. The inner temporal and inner nasal regions had the highest values, followed by the inner superior region. The mean ETDRS macula ring, inferior inner, superior inner, nasal inner, temporal inner, inferior outer, superior outer, nasal outer, temporal outer and central ring were: 83.4 ± 3.4, 83.9 ± 4.9, 84.6 ± 4.7, 84.8 ± 5.5, 83.4 ± 3.9, 77 ± 4.4, 79.8 ± 4.9, 78.8 ± 5.3, 77.6 ± 4.1, 102.5 ± 5.9 and 79.1 ± 6.4, 77.7 ± 9.4, 79.2 ± 7, 79.9 ± 7.5, 80.4 ± 5.7, 71.2 ± 8.6, 75.3 ± 8.1, 74.1 ± 7.3, 75.1 ± 6.9, 99.9 ± 10.6, from the control and HM groups respectively Table 2 and Fig. 3. Figure 1 illustrates an eye from control and HM groups, respectively.

Table 3 depicts the correlation between CT, ORL thickness, and RS with age, BCVA, SE, AL, and myopic maculopathy. Significantly, SE, AL, and myopic maculopathy emerged as the variables with the strongest and most frequent correlations with CT, ORL thickness, and RS.

**Table 3:** Correlation between choroidal thickness, outer retinal layer thickness and retinal sensitivity with age and other ocular parameters

Variable	Age, y		BCVA		SE		AL (mm)		Myopic maculopathy	
	r	p	r	p	r	p	r	p	r	p
Choroidal thickness (µm)										
Macula (mean ± SD)	− 0.13	0.271	− 0.09	0.445	0.492	** < 0.001**	0.538	** < 0.001**	− 0.539	** < 0.001**
II	0.151	0.198	0.087	0.462	0.546	** < 0.001**	0.489	** < 0.001**	− 0.54	** < 0.001**
SI	0.111	0.345	0.112	0.341	0.437	** < 0.001**	0.504	** < 0.001**	− 0.454	** < 0.001**
NI	0.065	0.584	0.089	0.453	0.453	** < 0.001**	0.538	** < 0.001**	− 0.51	** < 0.001**
TI	0.136	0.248	0.043	0.713	0.396	** < 0.001**	0.386	**0.001**	− 0.461	** < 0.001**
IO	0.155	0.188	0.097	0.413	0.479	** < 0.001**	0.539	** < 0.001**	− 0.545	** < 0.001**
SO	0.175	0.135	0.152	0.196	0.456	** < 0.001**	0.521	** < 0.001**	− 0.453	** < 0.001**
NO	0.019	0.876	0.037	0.756	0.443	** < 0.001**	0.606	** < 0.001**	− 0.526	** < 0.001**
TO	0.263	**0.024**	0.155	0.188	0.362	**0.002**	0.374	**0.001**	− 0.385	**0.001**
Central	0.086	0.464	0.013	0.913	0.445	** < 0.001**	0.526	** < 0.001**	− 0.529	** < 0.001**
ORL thickness (µm)										
Macula (mean ± SD)	0.314	**0.006**	0.276	**0.017**	0.399	** < 0.001**	0.431	** < 0.001**	− 0.418	** < 0.001**
II	0.226	0.053	0.115	0.329	0.348	**0.002**	0.509	** < 0.001**	− 0.393	**0.001**
SI	0.104	0.378	0.049	0.678	0.427	** < 0.001**	0.462	** < 0.001**	− 0.426	** < 0.001**
NI	− 0.22	0.06	0.143	0.223	0.421	** < 0.001**	0.471	** < 0.001**	− 0.348	**0.002**
TI	0.112	0.343	0.268	**0.021**	0.267	**0.022**	0.344	**0.003**	− 0.279	**0.016**
IO	− 0.14	0.235	0.202	0.085	0.364	**0.001**	0.411	** < 0.001**	− 0.38	**0.001**
SO	0.376	**0.001**	0.169	0.151	0.204	0.081	− 0.25	**0.032**	− 0.289	**0.013**
NO	0.235	**0.043**	0.131	0.267	0.344	**0.003**	0.395	** < 0.001**	− 0.404	** < 0.001**
TO	0.258	**0.026**	0.378	**0.001**	0.15	0.201	− 0.15	0.203	− 0.233	**0.046**
Central	0.304	**0.008**	0.281	**0.015**	0.13	0.271	0.153	0.194	− 0.129	0.275
Retinal Sensitivity (dB)										
Macula (mean ± SD)	0.218	0.062	0.021	0.858	0.287	**0.013**	0.435	** < 0.001**	− 0.348	**0.002**
II	0.355	**0.002**	0.325	**0.005**	0.219	0.061	0.403	** < 0.001**	− 0.366	**0.001**
SI	− 0.43	** < 0.001**	0.357	**0.002**	0.215	0.066	0.394	**0.001**	− 0.343	**0.003**
NI	0.326	**0.005**	0.206	0.079	0.366	**0.001**	0.523	** < 0.001**	− 0.48	** < 0.001**
TI	0.382	**0.001**	0.221	0.058	0.241	**0.038**	− 0.41	** < 0.001**	− 0.337	**0.003**
IO	0.334	**0.004**	0.166	0.159	0.201	0.085	0.359	**0.002**	− 0.287	**0.013**
SO	0.383	**0.001**	− 0.2	0.088	0.174	0.139	− 0.33	**0.004**	− 0.275	**0.018**
NO	0.251	**0.031**	0.145	0.217	0.341	**0.003**	0.505	** < 0.001**	− 0.406	** < 0.001**
TO	0.221	0.058	0.134	0.254	0.263	**0.024**	0.455	** < 0.001**	− 0.368	**0.001**
Central	0.439	** < 0.001**	0.488	** < 0.001**	0.26	**0.025**	0.371	**0.001**	− 0.322	**0.005**

Spearman’s coeficient correlation

Bold values represent the best statistically significant results (p < 0.05)

*BCVA* best correct visual acuity, *SE* spherical equivalent, *AL* axial length

The CT shows statistically significant changes based on age and axial length (p = 0.048 and p = 0.022, respectively). For every 10 year increase in the patient’s age, there was an average reduction of 14.4 μm in the CT, and for every one-unit increase in axial length, there was a reduction of 21.8 μm CT.

RS demonstrates a collectively statistically significant influence of both age and BCVA of the patient (p < 0.001 and p = 0.018, respectively). There is an average reduction of 1.6 dB for every 10 year increase in the patient’s age, and for every 0.1 logMar increase in BCVA, there is an average increase of 0.2 dB in RS.

According adjusted model, ORL thickness exhibits statistically significant changes based on the patient's age (p = 0.004). For each 10 years of age, there is an average reduction of 1.33 μm in retinal thickness.

**Fig. 4:**
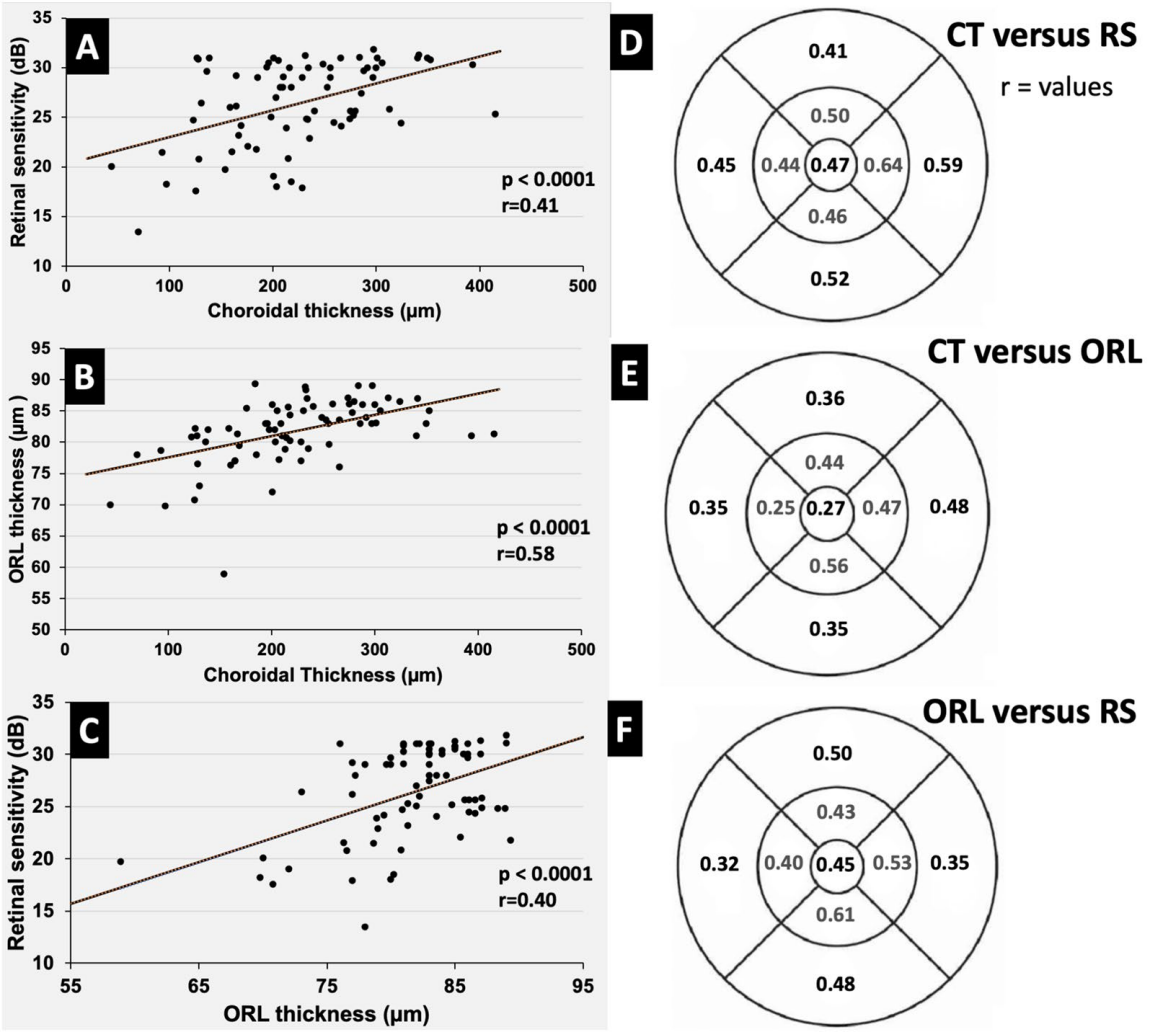
**A**–**C** scatter plot illustrating the mean macular correlation between retinal parameters, retinal sensitivity, outer retinal layer thickness and choroidal thickness. **D**–**F** discrimination of mentioned correlation is presented for each ETDRS sector (Sperman’s coefficient correlation)

Table 4 demonstrates the relationship between the three investigated parameters in this study. Both ORL thickness and RS are influenced by CT measurements (p < 0.001). For every 100-point increase in CT, there is an average increase of 3.4 μm in ORL thickness and 2.7 dB in RS. Figure 4 illustrates correlation in the macula and in each ETDRS region between CT, RS and ORL thickness.

**Table 4:** Relationship between macular (6 mmm) choroidal thickness and outer retinal layer thicknesses and retinal sensitivity

Variable	Factor	Coeficient	SE	95% CI of wald		Wald test	p
				Inferior	Superior		
ORL thickness	CT (µm)	74.14	1.78	70.651	77.62	1736.41	** < 0.001**
		0.034	0.0074	0.019	0.048	20.70	** < 0.001**
RS (dB)	CT (µm)	20.32	1.53	17.317	23.31	176.45	** < 0.001**
		0.027	0.0065	0.014	0.04	17.46	** < 0.001**

Generalized Estimating Equations (GEE) with normal distribution and identity-link function, assuming an exchangeable correlation matrix between the eyes

Bold values represent the best statistically significant results (p < 0.05)

*SE* spherical equivalente, *ORL* outer retinal layer, *RS* retinal sensitivity

In HM eyes, CT exhibited the highest area under the curve (AUC) in the receiver operating characteristic (ROC) analysis, measuring 0.8 (95% CI 0.7–0.9), compared to RS and ORL thickness, which measured 0.69 (95% CI 0.56–0.82) and 0.73 (95% CI 0.60–0.85), respectively.

CT, but not RS and ORL thickness, was the only tested parameter that presented significant correlation with maculopathy degree. According to Table 5, only the OCT showed a statistically significant influence on the classification of myopic maculopathy scale (p = 0.029), with an expected reduction of 1 point in the scale for every increase of 300 μm in the OCT.

**Table 5:** Models adjusted for myopic maculopathy classification according to choroidal thickness, retinal sensitivity and outer retinal layer

Factor	Coeficient	Standard error	Test statistic (wald)	p*
Constant	2.16	0.42	25.86	** < 0.001**
Choroidal thickness (µm)	− 0.0030	0.0015	4.75	**0.029**
Constant	2.00	0.61	10.76	**0.001**
Retinal sensitivity (dB)	− 0.0230	0.0214	1.11	0.292
Constant	3.25	2.09	2.42	0.120
ORL thickness (µm)	− 0.0230	0.0253	0.80	0.372

Bold values represent the best statistically significant results (p < 0.05)

*Generalized Estimating Equations (GEE) with Poisson distribution

*ORL* outer retinal layer

## Discussion

Our results revealed a reduction in volumetric automated CT measurements in patients with HM, in accordance with previous studies [17]. This thinning may be caused from elongation-induced mechanical stretching of the choroid, potentially leading to choriocapillaris and choroidal vessel occlusion and the subsequent replacement of normal choroidal structure with fibrous tissue [18–20].

Our study revealed that automated CT measurements vary significantly depending on their location. Specifically, the CT at the superior region in the macula was identified as the thickest region, a finding consistent with previous publications, even when utilizing manual single measures. Conversely, the nasal CT was observed as the thinnest region, followed by the central foveal and inferior choroid, and then the temporal region. This is an intriguing finding since there are only speculation about the cause of this pattern. The most plausible explanation would be the embryologic influence, as the closure of the optic fissure around 7 weeks of gestation [21] may result in significant thinning, particularly in the inferior part of the myopic eye. However, this explanation may not entirely account for the distinctive thickness pattern, given that the in this study inferior choroid is not typically the thinnest macular region, even in nonmyopic eyes. The second hypothesis would be the influence by the watershed zone of the choroid, commonly recognized as a hypofilling area in choroidal angiography [22].

Given the choroid’s role in nourishing photoreceptors, [23] the observed choroidal thinning in HM eyes could potentially impact retinal function. Our study noted reduced RS, as measured by microperimetry, in the HM group, aligning with findings from prior study [13]. This decline in sensitivity may be attributed to the compromised structural integrity of retinal layers in highly myopic eyes [24]. Likely a consequence of the stretching of the eye globe.

This study is unprecedented in its use of the ETDRS map, allowing for a precise and direct topography correlation between macular regions and their structural components with RS. A moderate direct correlation was observed between CT and RS in the HM group, a finding consistent with prior study [24]. While it might be anticipated that a thicker choroid would contribute to improved retinal function and RS, our study, similar to the findings of Zaben et al., revealed that the superior ETDRS region was not the site with the highest RS values or the region of lowest sensitivity in HM eyes.

Interestingly, our study identified the outer nasal ETDRS grid as the location with the lowest RS value and the inner temporal as the region with the highest value. Despite both our study and Zaben’s showing a direct proportional correlation between RS and CT, neither demonstrated greater sensitivity in the superior macular region where the choroid is thicker in HM [13]. Hence, there may be other factors influencing the distribution of RS within the macular region.

Analyzing the photoreceptors present in the ORL could potentially provide insights into this matter, our study also revealed thinning of the ORL in HM eyes, a result also consistent with prior studies [25, 26]. The likely etiology may be attributed to biomechanical changes and stretching of the retinal tissue due to the increased axial length seen in myopia [27].

It is crucial to recognize that thinning in this layer can have implications for visual function and, consequently, the quality of life of individuals. This study observed that patients with HM presented decreased foveal BCVA and RS, aligning with prior investigations [24]. Similar to RS, ORL thinning did not follow the pattern of macular CT, and the superior macula was not the region of thicker ORL even though ORL thickness demonstrated positive correlation with CT. Some regions, such as the nasal macula, seem to align in terms of parameter values, as it was the region of thinnest CT, thinner ORL, and low RS. The possible reason for this could be that a healthier, thicker choroid may provide better support and nourishment to the outer retina. There might be a threshold where the thickness of the choroid can impact ORL thickness and RS, leading to subsequent damage to visual function.

When evaluating structural and functional retinal alterations, using the area under the curve, choroidal thickness (CT) stands out as a prominent factor, demonstrating superior diagnostic performance in identifying high myopic (HM) eyes compared to retinal sensitivity (RS) and outer retinal layer (ORL) thickness. Notably, CT was the sole factor that had a noticeable impact on myopic maculopathy classification when the model was adjusted.

However, it is crucial to acknowledge the limitations of our study. The most notable limitation was the relatively small sample size, consisting of 37 patients, even though the statistical analysis was conducted on 74 eyes, potentially limiting the generalizability of our findings. Additionally, the manual measurement of ORL thickness represents another limitation in our study.

## Conclusion

High myopia is related to ORL and choroidal changes as well as retinal sensitivity. Choroidal thinning contributes to reduced retinal sensitivity, ORL thinning, and an exacerbation of myopic maculopathy degree. Despite positive correlations between choroidal thickness (CT), ORL thickness, and retinal sensitivity (RS), the superior macular region, where the choroid is thicker, does not necessarily correspond to the region with the greatest RS or thicker ORL in patients with high myopia. Hence, there is a global need for innovative treatments to prevent the evolution of myopia and its negative impact on visual function.

## Data Availability

The data that support the findings of this study are under the policy of the institution and available from the corresponding author on reasonable request.

## References

[1] Ba H, Tr F, Da W, MKSP JNS (2016). Global prevalence of myopia and high myopia and temporal trends from 2000 through 2050. Ophthalmology.

[2] Cao J, McLeod S, Merges CA, Lutty GA (1998). Choriocapillaris degeneration and related pathologic changes in human diabetic eyes. Arch Ophthalmol.

[3] Spaide RF (2009). Age-related choroidal atrophy. Am J Ophthalmol.

[4] Jb J, L X (2014). Histological changes of high axial myopia. Eye.

[5] Fujiwara T, Imamura Y, Margolis R, Slakter JS, Spaide RF (2009). Enhanced depth imaging optical coherence tomography of the choroid in highly myopic eyes. Am J Ophthalmol.

[6] Ikuno Y (2017). Overview of the complications of high myopia. Retina.

[7] Borges FB, Zacharias LC, Pimentel SLG, Cunha LP, Monteiro MLR, Preti RC (2022). Morphofunctional evaluation of peripapillary retinoschisis associated with myopic posterior staphyloma and hyaloid traction: does it cause peripapillary vitreoretinal traction?. Arq Bras Oftalmol.

[8] Photocoagulation for diabetic macular edema (1985). Photocoagulation for diabetic macular edema early treatment diabetic retinopathy study report number 1 early treatment diabetic retinopathy study research group. Arch Ophthalmol.

[9] Adhi M, Duker JS (2013). Optical coherence tomography—current and future applications. Curr Opin Ophthalmol.

[10] Laishram M, Srikanth K, Rajalakshmi AR, Nagarajan S, Ezhumalai G (2017). Microperimetry—a new tool for assessing retinal sensitivity in macular diseases. J Clin Diagn Res.

[11] Matsuura M, Murata H, Fujino Y, Hirasawa K, Yanagisawa M, Asaoka R (2018). Evaluating the usefulness of MP-3 microperimetry in glaucoma patients. Am J Ophthalmol.

[12] Qin Y, Zhu M, Qu X, Xu G, Yu Y, Witt RE (2010). Regional macular light sensitivity changes in myopic Chinese adults: an MP1 study. Invest Ophthalmol Vis Sci.

[13] Zaben A, Zapata M, Garcia-Arumi J (2015). Retinal sensitivity and choroidal thickness in high myopia. Retina.

[14] Zengin MO, Cinar E, Kucukerdonmez C (2015). International photographic classification and grading system for myopic maculopathy. Am J Ophthalmol.

[15] Zengin MO, Cinar E, Kucukerdonmez C (2014). The effect of nicotine on choroidal thickness. Br J Ophthalmol.

[16] Germano RAS, Hatanaka Ma, Junior RS (2016). Choroidal thickness variation in highly myopic eyes during the water drinking test. Arq Bras Oftalmol.

[17] Ikuno Y, Tano Y (2009). Retinal and choroidal biometry in highly myopic eyes with spectral-domain optical coherence tomography. Invest Ophthalmol Vis Sci.

[18] Duke-Elder SAD (1970). Pathological myopia Duke–Elder’s system of ophthalmology. refractive optics and refraction.

[19] Ohno H (1983). Electron microscopic studies of myopic retinochoroidal atrophies 1 choroidal changes. Fol Ophthalmol Jpn.

[20] Matsuo NOS, Hasegawa E, Okamoto S (1978). Report of research, Tokyo: cocdiJ.

[21] Cw O (1999). The human eye: structure and function.

[22] Hayreh SS (1990). In vivo choroidal circulation and its watershed zones. Eye.

[23] Nickla DL, Wallman J (2010). The multifunctional choroid. Prog Retin Eye Res.

[24] Park UC, Yoon CK, Bae K, Lee EK (2022). Association of retinal sensitivity with optical coherence tomography microstructure in highly myopic patients. Invest Ophthalmol Vis Sci.

[25] Ye J, Shen M, Huang S, Fan Y, Yao A, Pan C (2019). Visual acuity in pathological myopia is correlated with the photoreceptor myoid and ellipsoid zone thickness and affected by choroid thickness. Invest Ophthalmol Vis Sci.

[26] Wang Y, Ye J, Shen M, Yao A, Xue A, Fan Y (2019). Photoreceptor degeneration is correlated with the deterioration of macular retinal sensitivity in high myopia. Invest Ophthalmol Vis Sci.

[27] Wu PC, Chen YJ, Chen CH, Chen YH, Shin SJ, Yang HJ (2008). Assessment of macular retinal thickness and volume in normal eyes and highly myopic eyes with third-generation optical coherence tomography. Eye.

